# Genetic Disorders Associated with Metal Metabolism

**DOI:** 10.3390/cells8121598

**Published:** 2019-12-09

**Authors:** Muhammad Umair, Majid Alfadhel

**Affiliations:** 1 Medical Genomics Research Department, King Abdullah International Medical Research Center (KAIMRC), King Saud Bin Abdulaziz University for Health Sciences, Ministry of National Guard Health Affairs (MNGH), P.O Box 3660, Riyadh 11481, Saudi Arabia; khugoo4u@yahoo.com; 2 Division of Genetics, Department of Pediatrics, King Abdullah Specialized Children’s Hospital, King Abdulaziz Medical P.O Box 22490, Riyadh 11426, Saudi Arabia

**Keywords:** metal metabolism, genetic disorders, copper, iron, manganese, zinc, selenium

## Abstract

Genetic disorders associated with metal metabolism form a large group of disorders and mostly result from defects in the proteins/enzymes involved in nutrient metabolism and energy production. These defects can affect different metabolic pathways and cause mild to severe disorders related to metal metabolism. Some disorders have moderate to severe clinical consequences. In severe cases, these elements accumulate in different tissues and organs, particularly the brain. As they are toxic and interfere with normal biological functions, the severity of the disorder increases. However, the human body requires a very small amount of these elements, and a deficiency of or increase in these elements can cause different genetic disorders to occur. Some of the metals discussed in the present review are copper, iron, manganese, zinc, and selenium. These elements may play a key role in the pathology and physiology of the nervous system.

## 1. Introduction

Metal ions play an important role in several biological processes that have both structural and functional importance. Human beings gain an adequate amount of nutrient metals from their daily diet, but need to avoid a toxic or an excess amount of metals for an ideal health. Both an imbalance in the amount of nutrient metals in the body and exposure to toxic metals are associated with severe health problems. They are linked to several abnormalities, including cardiovascular diseases, neurodegenerative disorders (Alzheimer’s disease, Parkinson’s disease, and Huntington’s disease), and metabolic disorders [1,2,3,4,5]. The toxicity of and pathways involved in metal imbalances are now considered to be highly active areas of biomedical research. The findings of these studies might inspire us to develop new classes of therapeutic strategies and help us to understand metal metabolism [5,6].

### 1.1. Transfer of Groups

“Transitional elements” or “transitional metals” are those elements that are present in the d-series (Groups 3 to 12) of the periodic table. All of the transitional or trace metals also participate in reactions that involve the transfer of specific groups. These include either phosphorylation (when a phosphate residue is transferred) or dephosphorylation (when a phosphate residue is removed). Similarly, glycosylation is the process by which a sugar residue is transferred [7].

### 1.2. Neurotoxicity

Neurotoxicity results in the accumulation of elements in the brain, such as copper, selenium, iron, and manganese, and can lead to different types of brain-associated disorders. Specific parts of the brain, such as the substantia nigra and the basal ganglia, are predominantly exposed to such changes. Some of the metals discussed in the present review are copper, iron, manganese, zinc, and selenium, which play a very important role in human biology. Several disorders that involve an accumulation of different metal elements in the human brain have been reported in the literature and are discussed in the present review [4,8,9,10,11,12]. Some disorders that are due to an accumulation of manganese, copper, or iron and cause neurotoxicity are summarized in Table 1. The present review will highlight: (i) Disorders associated with zinc homeostasis; (ii) disorders associated with iron homeostasis; (iii) disorders associated with copper homeostasis; (iv) disorders associated with manganese homeostasis; and (v) disorders associated with selenium homeostasis.

## 2. Zinc Homeostasis

**Figure 1:**
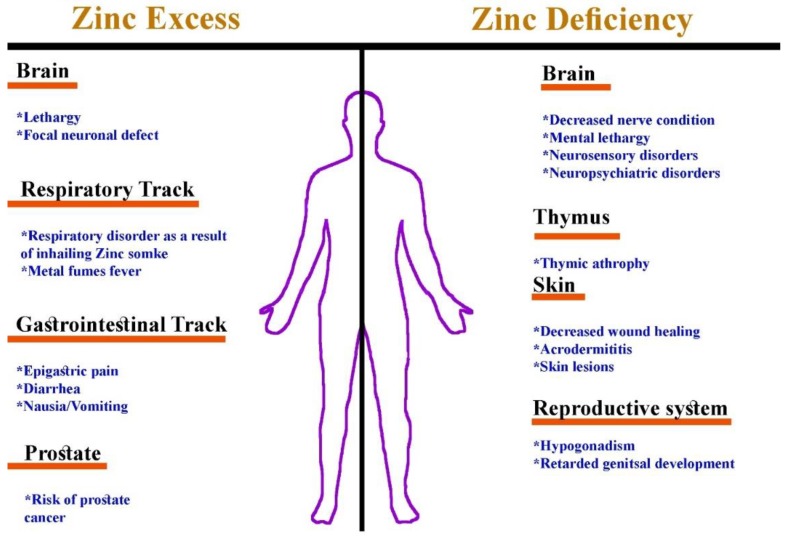
A schematic representation of zinc deficiency as compared to zinc intoxication.

Zinc plays a very important role in the human body and acts as a cofactor for several enzymes and transcription factors that contain modules for binding DNA and RNA (zinc fingers), including alkaline phosphatase, lactate dehydrogenase, ornithine transcarbamylase, superoxide dismutase, carbonic anhydrase, DNA and RNA polymerases, matrix metalloproteinases, insulin-degrading enzymes, N-acyl D-aspartate acylase, and alcohol dehydrogenase [13]. Zinc metalloenzymes are involved in the regulation of different metabolic pathways and have structural, catalytic, and regulatory functions [14]. Although zinc is important to proper regulation of several important physiological functions, both an excess and a deficiency of zinc have been associated with several conditions (Figure 1).

**Figure 2:**
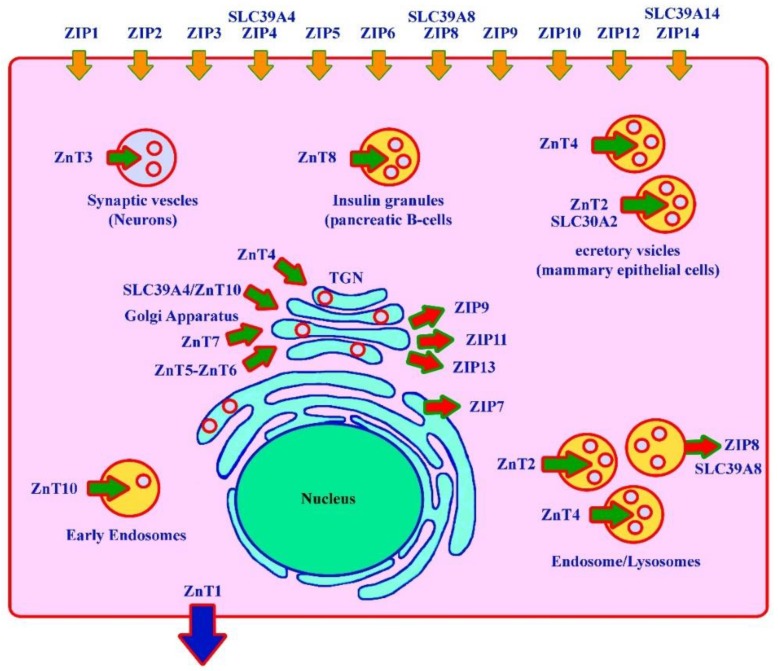
*SLC39A* family members (zinc/iron-regulated transporter-like protein (ZIP) transporters) and their role at the cellular level. Zn transporters belong to the *SLC30A* family. These cause different disorders in humans.

Several members of the *SLC39A* family encode for different zinc/iron-regulated transporter-like protein (ZIP) transporters (ZIP1–6, 8–10, 12, and 14). Their main function is the uptake of zinc at the cell surface; however, some family members also have the ability to transport manganese, iron, and cadmium [15]. Similarly, it has been reported that both *SLC39A14* and *SLC39A8* have similar functions and mediate manganese uptake at the cell surface [16]. Deficiencies in these ZIP transporters result in different disorders in humans (Figure 2). Many disorders have been associated with zinc deficiency; however, only a few are discussed here.

### 2.1. Disorders Associated with Zinc Homeostasis

#### 2.1.1. Acrodermatitis Enteropathica

Acrodermatitis enteropathica (OMIM 201100) is an autosomal recessive disorder caused by biallelic sequence variants in the *SLC39A4* gene (OMIM 607059) located on chromosome 8q24. The *SLC39A4* (Solute Carrier Family 39 Member 4) gene encodes for ZIP4, which belongs to the zinc/iron-regulated transporter-like protein (ZIP) family. The clinical description includes short stature, failure to thrive, hepatomegaly (liver), decreased intestinal uptake of zinc, diarrhea, poor appetite, bullous pemphigoid, pustular dermatitis on extremities, necrosis with inflammation, alopecia, cerebellar ataxia, decreased testosterone in males, and a decrease in plasma zinc, serum alkaline phosphatase, and mucosal alkaline phosphatase levels [4,17,18,19,20]. To date, only 52 disease-causing mutations associated with acrodermatitis enteropathica have been reported in the *SLC39A4* gene (HGMD, 2018; http://www.hgmd.cf.ac.uk).

#### 2.1.2. Transient Neonatal Zinc Deficiency

Transient neonatal zinc deficiency (OMIM 608118) is an autosomal dominant disorder caused by heterozygous or de novo sequence variants in the *SLC30A2* gene (OMIM 609617) located on chromosome 1p36.11. The *SLC30A2* (Solute carrier family 30 member 2) gene encodes for ZNT2, which is a zinc transporter that acts as a homodimer in mammary epithelial cells. This disorder leads to low zinc concentrations in maternal milk and the infant might show clinical phenotypic similarity to acrodermatitis enteropathica, including dermatitis and partial alopecia [21,22,23]. To date, only 28 disease-causing mutations associated with zinc deficiency in breast milk have been reported in the *SLC30A2* gene (HGMD, 2018; http://www.hgmd.cf.ac.uk).

#### 2.1.3. Ehlers-Danlos Syndrome (Spondylodysplastic Type 3: SCD-EDS)

Biallelic mutations in the *SLC39A13* gene (OMIM 608735) located on chromosome 11p11.2, which encodes for the zinc transporter ZIP13, causes severe SCD-EDS (OMIM 612350). It is characterized by an EDS-like phenotype, including short stature, protuberant eyes, bifid uvula, a high palate, hypodontia, delayed teeth eruption, muscle atrophy, platyspondyly, osteopenia, joint laxity, flat feet, irregular vertebral bodies, and other skeletal manifestations [24].

Usually, the serum zinc levels are normal but the intracellular zinc distribution is abnormal, leading to an abnormal nuclear translocation of SMADs. This results in alterations in BMP and TGFβ signaling in collagen-producing cells [25].

#### 2.1.4. Birk-Landau-Perez Syndrome

Birk-Landau-Perez syndrome is an autosomal recessive condition caused by homozygous mutations in the *SLC30A9* gene located on chromosome 4p13. The *SLC30A9* (Solute carrier family 30 member 9) gene encodes for the 586 amino-acid-long ZnT9 (Zinc transporter 9) protein. ZnT9 is localized in the endoplasmic reticulum (ER), and a ZnT9 shortage leads to interrupted zinc homeostasis and a decrease in zinc cytosolic concentrations. The clinical characterization includes oculomotor apraxia, abnormal eye movements, ptosis, renal dysfunction, hyperechogenic kidneys, axial hypotonia, cognitive impairment, dyskinesia, choreoathetosis, dystonia, and hyperkalemia [26]. To date, only a single Arab family from Bedouin has been reported as having Birk-Landau-Perez syndrome. Using genome-wide homozygosity mapping and whole exome sequencing, Perez et al. (26) reported a frame shift variant (c.1047_1049delGCA; p.A350del) in the *SLC30A9* gene.

## 3. Iron Homeostasis

In humans, iron is absorbed through the intestinal tract and excreted through the urine and the bile. In healthy adults, the normal iron pool ranges from 2 to 6 g, 0.5 g of which is usually stored in the liver, and includes 98% hepatocytes [27,28]. Iron absorption requires specific transporters that are located in the basolateral and apical membranes of the intestinal mucosal cells. Ferroportin, which is a basolateral transporter, can bind to the hepcidin protein, which inactivates the ferroportin and restricts iron absorption in the mucosal cells. High transferrin saturation results in the release of hepcidin in the liver in response, which inhibits iron release from macrophages. Whenever the absorption of iron is excessive, it results in an iron overload [29].

**Figure 3:**
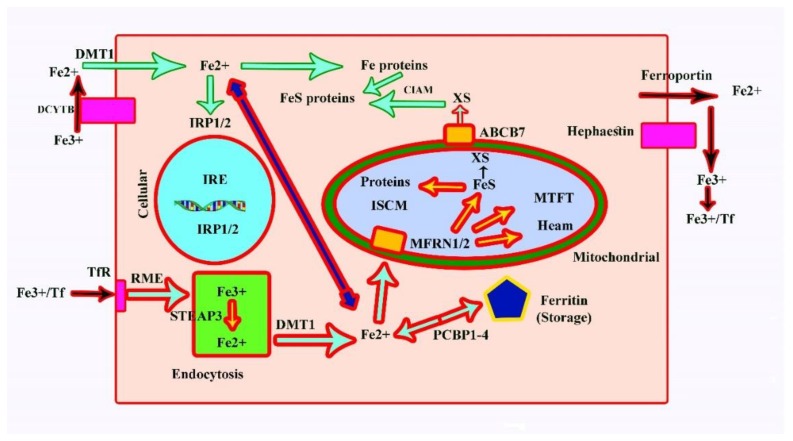
Iron metabolism at the cellular level and its regulation. TfR: Transferrin receptor; IRE: Iron-responsive element; RME: Receptor-mediated endocytosis; E: Endosome; ISCM: Iron–sulphur cluster machinery; Ff: Transferrin; IRP: Iron-responsive protein; FeS: Iron–sulphur cluster; XS: Unknown sulphur transporter; CIAM: Cytosolic iron–sulphur cluster assembly machinery; PCBP: Poly (rC) binding protein.

Transcription of hepcidin results from an increase in the iron level in plasma, which has major effects on such target cells as macrophages and principally enterocytes (Figure 3). In both of these cells, an increase in hepcidin causes the degradation of ferroportin, which exports iron from the cell. The increase in hepcidin will affect macrophages, resulting in a reduced amount of iron being released into the plasma. To bring the amount of iron back into a normal range, the net amount of iron in the plasma needs to be reduced [28,29].

Iron from the plasma is taken up by cells only if the hepcidin pathway is impaired. These cells include those of the pancreas, liver, heart, and several other organs that may cause diabetes, cirrhosis, and cardiomyopathy. Hepcidin levels can also be triggered in the case of inflammation and infection until the amount of iron in the plasma returns to normal, which leads to a low amount of iron being observed in the plasma during chronic infection/inflammation [29]. Mutations in genes responsible for causing genetic haemachromatosis are listed in Table 2. These include *HAMP, HFE1, HJV, HAMP, TRF2*, and *SLC40A1*.

### 3.1. Cellular Processing of Iron and Its Regulation

Iron (Fe2+) uptake at the surface of cells occurs with the help of DMT1, which encodes a divalent metal transporter that also helps uptake into enterocytes. As a result, the Fe3+-bound transferrin binds the transferrin receptor at the surface of the cell and results in endocytosis [17,30]; Figure 3).

First, the uptake (by endocytotic vesicles) requires the conversion of Fe3+ to Fe2+, which is done by the endosomal ferrireductase STEAP3 followed by DMT1. Mitoferrin 1 (MFRN1; *SLC25A37*) and mitoferrin 2 (MFRN2; *SLC25A28*) are required to transport iron into the mitochondria. Mitochondrial Fe2+ has two principal destinies, including synthesis of iron–sulphur clusters and synthesis of haem. Within mitochondria, iron is normally stored as mitochondrial ferritin (MTFT). Cytosolic Fe can be converted into FeS proteins and Fe proteins with the help of the PCB1-4-dependent pathway into ferritin [17,30].

Proper regulation of the iron homeostasis in the cytosole comprises binding of iron to iron-responsive proteins 1/2 (IRP1/2), which are also designated as iron-responsive element binding proteins (IREB1/2). These IRPs bind to IREs and control transcription of the iron-responsive proteins. The inability of the cell to use iron for haem group synthesis leads to the form of iron deficiency known as anaemia and can cause an overload of iron in the liver. The disorders that result due to iron overload are listed in Table 3 [31,32].

### 3.2. Iron Homeostasis and Related Disorders

#### 3.2.1. Ferroportin Disease

Pathogenic heterozygous mutations in the *SLC40A1* gene (OMIM 604653) cause hemochromatosis type 4 (OMIM 606069). *SLC40A1* encodes for ferroportin located on chromosome 2q32.2, which is the only known iron exporter in mammals. *SLC40A1* loss-of-function (LOF) mutations lead to the classical type A ferroportin disease, which is characterized by such features as cataracts, cardiomyopathy, fibrosis, joint pains, osteoarthritis, hyperpigmentation, endocrine disorders, and anemia [33,34].

#### 3.2.2. Hereditary Hemochromatosis

Parenchymal cells in the liver and pancreas store most of the body’s iron, and an excessive accumulation of iron may lead to hemochromatosis [35]. In this condition, the total amount of accumulated iron exceeds more than 50 g in the body. This accumulation of iron is life long and usually takes five to six decades to show symptoms [36,37]. Mutations have been reported in five different genes associated with hemochromatosis, including *HJV* (HFE) and *HAMP*. Both are responsible for causing hemochromatosis type 2, also known as juvenile hemochromatosis (OMIM 602390), which results in severe iron overload at <30 years of age. Mutations in the *TFR2* gene cause hemochromatosis type 3 (OMIM 604250), and *FTH1* heterozygous mutations result in hemochromatosis type 5 (OMIM 615517).

All of these gene products function in pathways that are involved in the production of hepcidin in the hepatocytes. Thus, a lack of proper hepcidin production results in disrupted regulation of iron absorption in the intestinal track.

#### 3.2.3. Neurodegeneration with Iron Accumulation in the Brain (NBIA)

In the literature, several inborn brain iron accumulation disorders have been reported [38]. NBIA comprises a group of neurodegenerative diseases that are mostly characterized by excessive iron accumulation in different parts of the central nervous system (CNS), particularly in the basal ganglia [38,39]. The two most common types that result due to iron homeostasis are discussed here. A list of genes involved in NBIA is presented in Table 3.

#### 3.2.4. Acaeruloplasminaemia

Homozygous variants in the *CP* gene cause unnoticeable levels of caeruloplasmin in the plasma. Affected individuals have excess iron accumulated in the islets of the Langerhans (liver) and the brain. Affected individuals suffer from neurological symptoms (ataxia, chorea, dystonia, psychiatric disorders, and Parkinsonism), diabetes mellitus, and retinal degeneration. Ferrous (Fe2+) iron is converted into ferric (Fe3+) iron in the plasma by an enzyme called caeruloplasmin, which is a copper-containing enzyme secreted by the liver. Affected individuals suffering from acaeruloplasminaemia have high serumferritin and low serum iron levels [40,41]. To date, only 60 disease-causing mutations associated with acaeruloplasminaemia and Parkinsonism-related disorders have been reported in the *CP* gene (HGMD, 2018; http://www.hgmd.cf.ac.uk).

#### 3.2.5. Neuroferritinopathy

Heterozygous variants in the *FTL* gene (OMIM 134790) cause hyperferritinemia cataract syndrome (OMIM 60886) and neurodegeneration with brain iron accumulation type 3 (OMIM 606159). Associated clinical phenotypes include blepharospasm, palatal tremor, dystonia, micrographia, dysphagia, gait disability, tremor, Parkinsonism, choreoathetosis, and hyper-reflexia [42,43]. To date, only 64 disease-causing mutations associated with hyperferritinemia cataract syndrome have been reported in the *FTL* gene (HGMD, 2018; http://www.hgmd.cf.ac.uk).

## 4. Copper Homeostasis

Approximately 100 mg of copper is stored in the human body, of which 35 mg is contained in muscles, 10 mg is contained in connective tissues, 20 mg is contained in the brain, 10 mg is contained in the blood, and 5 mg is contained in the kidneys [44,45]. Approximately 1–5 mg of copper is excreted from and absorbed into the human body daily, such that the net balance is zero (Table 4). Copper in the plasma, which accounts for 90%–95%, is bound to an alpha-glycoprotein ceruloplasmin, and the binding process is completed in the liver once it is hepatically taken up. Different copper-containing enzymes are listed in Table 5, which also includes dopamine hydroxylase and tyrosine hydroxylase. These two produce neurotransmitters in the CNS. The list also includes an enzyme cytochrome c oxidase, which is very important to mitochondrial energy production, electron transport, and proteins related to ceruloplasmin, a blue-colored plasma protein that binds up to 95% of circulating copper and plays a key role in the regulation of iron homeostasis [46,47,48].

### 4.1. Disorders Associated with Copper Homeostasis

#### 4.1.1. Wilson Disease

Wilson disease (OMIM 277900) is an autosomal recessive inherited disorder characterized by buildup of intracellular hepatic copper with severe neurological and hepatic abnormalities. It is caused by biallelic mutations in the *ATP7B* gene (OMIM 606882) located on chromosome 13q14 and encodes a cation-transporting, P-type adenosine triphosphatase (ATPase). Associated clinical features include a Kayser-Fleischer ring, hepatic cirrhosis, comma, liver failure, hepatocellular carcinoma, renal tubular dysfunction, renal calculi, osteoporosis, osteoarthritis, joint hypermobility, dysphagia, dementia, dystonia, and tremors [49].

The disease has a prevalence of 10/3000 live births and the heterozygote-carrier state has a prevalence of slightly higher than 1/100. Wilson disease is very common in consanguineous populations, such as certain isolated communities in Sardinia, Japan, and the Middle East. Although heterozygous individuals are phenotypically normal, approximately 20% have low serum copper and ceruloplasmin levels [50,51,52,53,54,55].

More than 700 mutations causing Wilson disease have been reported in the *ATP7B* gene, and almost half of these are missense mutations (H1069Q, most common) [56]; HGMD, 2018). The protein that is encoded by the *ATP7B* gene functions in the trans-Golgi region of hepatocytes and transports copper into the secretory system. A homozygous mutation in the *ATP7A* gene results in a failure to uptake copper, resulting in a cellular copper deficiency. This copper deficiency gives rise to three main phenotypes: Menkes disease, X-linked distal neuropathy, and occipital horn syndrome [57]. Mutations that lead to improper copper excretion result in copper toxicity, which leads to Wilson disease and affects the liver and central nervous system.

##### Neurological Presentation in Wilson Disease

Individuals affected with Wilson disease manifest neurological symptoms within 8–20 years of age. Patients also have a movement disorder, such as dysarthria, dysgraphia, tremor (progressive), drooling, and dystonics. MRI and a CT scan are typically used to visualize the changes in the striatum; however, patients show different densities due to clinical heterogeneity [58]. The phenotype can become severe while under treatment, with only 34% of patients showing improvement in these symptoms. It has been reported that 35% of patients treated with D-penicillamine and 19% treated with zinc sulphate suffered from worse neurological symptoms [59,60]. However, neurological deterioration was reported in 27% of affected individuals treated with trientine [54,55]. Further research is required to find an appropriate drug to treat such a severe neurological disorder.

##### Liver Failure Associated with Wilson Disease

Liver failure is a life-threatening issue for patients with Wilson’s disease, and, currently, if the severity score (SS) is 11, the only treatment is liver transplantation [61]. This is very difficult to accomplish due to donor organ availability and matching. It has been reported that, in an ATP7B-deficient rat, methanobactin treatment reversed acute liver failure. Methanobactin is reported to have a high affinity to copper and to be more efficient in extracting copper in *Atp7b^-/-^* knockout rats [62].

#### 4.1.2. MEDNIK Syndrome 

MEDNIK syndrome (OMIM 609313) is caused by mutations in the *AP1S1* gene (OMIM 603531) located on chromosome 7q22.1. The *AP1S1* gene encodes adaptor protein complex 1 subunit β1, which is necessary for the trafficking of ATP7 (A and B) out of the trans-Golgi network (TGN). The clinical phenotype associated with MEDNIK syndrome includes enteropathy, mental retardation, deafness, keratoderma, peripheral neuropathy, brain atrophy, ichthyosis, and cholestatic hepatopathy [63]. In this disorder, the caeruloplasmin and copper concentrations are elevated in the liver and reduced in the plasma [63,64]. To date, only three disease-causing mutations associated with *AP1S1*-related MEDNIK syndrome have been reported in the literature (HGMD, 2018; http://www.hgmd.cf.ac.uk).

#### 4.1.3. Menkes Disease

Menkes disease (OMIM 309400) is a copper deficiency disorder inherited in an X-linked recessive fashion and mutations in the *ATP7A* gene (OMIM 300011) have been associated with this disorder. *ATP7A* (ATPase Copper Transporting Alpha) is a protein-coding gene located on chromosome Xq21.1. The clinical features associated with Menkes disease include microcephaly, short stature, intracranial hemorrhage, osteoporosis, skin hypopigmentation, hypotonia, mental retardation, seizures, and low copper and ceruloplasmin levels upon laboratory analysis. To date, more than 350 mutations responsible for Menkes disease in the *ATP7A* gene have been reported (HGMD, 2018; http://www.hgmd.cf.ac.uk). It is characterized by the proper transport of copper into copper-containing enzymes, which results in low ceruloplasmin and copper serum levels [65,66,67,68,69]. The incidence of Menkes disease is approximately 10/25,000 live births [67].

#### 4.1.4. Occipital Horn Syndrome (OHS)

OHS is an X-linked recessive disorder characterized by cutis laxa, coarse hair, cerebral calcification exostoses, hyperextensible skin, mild cognitive deficits, global developmental delay, and loose joints. This condition might affect copper levels in the body and is regarded as a mild form of Menkes disease. OHS is caused by pathogenic disease-causing mutations in the *ATP7A* gene (OMIM 300489) [70,71,72]. The *ATP7A* gene encodes a P-type ATPase whose function is to transport copper across membranes. The ATP7A protein is mostly localized in the trans Golgi, where it helps to supply copper to different enzymes that are copper-dependent in the secretory pathway [72,73].

#### 4.1.5. Huppke-Brendel Syndrome (HBS)

HBS is a recessive disorder characterized by bilateral congenital cataracts, developmental delay, cerebral atrophy, hypacusis, hearing loss, and nystagmus. MRI of the brain reveals cerebellar hypoplasia, hypomyelination, and wide subarachnoid spaces. To date, only a few patients have been reported as having low serum copper and ceruloplasmin levels [74,75]. It is caused by homozygous or compound heterozygous mutations in the *SLC33A1* gene (OMIM 603690) located on chromosome 3q25.31.

#### 4.1.6. X-Linked Distal Hereditary Motor Neuropathy

Distal Hereditary Motor Neuropathy is a rare X-linked disorder characterized by such features as progressive weakness of distal muscles, motor neuron syndrome, affected peripheral nerves, muscle atrophy, and an abnormal sensory examination [76]. Genetic analysis revealed a maximum LOD score of 3.46 at Xq13.1-q21, flanked by markers DXS8052 and DXS559. Sequencing of the *GJB1* gene and its promoter did not reveal any mutation. Later, a DSMAX locus was found to be flanked by the DXS8046 and DXS8114 markers (in the 14.2 Mb region) [76].

#### 4.1.7. Alzheimer’s Disease and Copper

Alzheimer’s disease (AD) is a progressive neurodegenerative disease characterized by development of senile plaques in the brain [77]. High levels of polyvalent cations (copper, iron, and zinc) are observed in the senile plaques in the brains of AD patients [78,79,80]. Mouse models were used to reveal that copper accumulates in the senile plaques of AD patients with neurodegeneration, however, in a PSAPP (presenilin/amyloid precursor protein) mouse model with slight neurodegeneration, no copper accumulation was observed [81]. Thus, these data highlight that copper and zinc ions are not the only culprits associated with AD pathogenesis [82]. A study revealed that abnormal copper homeostasis with an increase in the copper pool and a decrease in copper-bound proteins are the main factors causing AD [83].

Furthermore, an increased serum copper concentration in patients with AD might be associated with an increase in the concentrations of “free” copper in the blood [83,84]. Mutations in genes encoding for proteins required for copper ion uptake lead to early onset familial AD [85]. This highlights that a copper deficiency in the brain cells seems to be an important factor in AD [77].

#### 4.1.8. Parkinson’s Disease and Copper

There are several important pieces of evidence in the literature concerning the role of free copper in neurodegenerative diseases [86,87]. Occupational studies have noted that long-term exposure (20 years) to copper and manganese increases the risk of Parkinson’s disease [88]. In an in vitro study, Spencer et al. (89) demonstrated that copper ions facilitate the oxidation of dopamine and other related catechols, such as 6OH-DOPA and L-dopamine. The complexes resulting from dopamine oxidation products and copper were observed to cause intense damage to DNA [89]. Copper is required in a myriad of reactions in cell metabolism, mainly in the brain because the brain is prone to oxidative stress and has a high respiratory rate [90]. In this regard, an important physiological function of copper-dependent proteins is their redox capacity. The role of copper transporters in Parkinson’s disease is an issue that also deserves further research [91]. Knowledge about copper compartmentalization in the brain will help to establish promissory therapeutic strategies aimed at enhancing the positive role of this metal in Parkinson’s disease [92].

## 5. Manganese Homeostasis

Manganese (Mn) is a heavy metal required for several important physiological processes in humans, such as energy metabolism, antioxidant defense, and proper immune function. However, overexposure to or an excess amount of Mn can result in a condition that may be similar to Parkinson’s disease (PD). In humans, the total required amount of manganese is 12–20 mg. The daily turnover is estimated to be 20 μg, with approximately 2–22 mg/day as the intake, and approximately 2%–10% absorbed through the small intestine [16]. There is a competition between the daily iron and manganese intake in the body, as these elements compete for the same divalent metal transporter, DMT1 [16,93]. Enzymes that require Mn for proper function are presented in Table 6.

In several studies, *Caenorhabditis elegans* has been shown to mimic the effects of excess manganese in mammals, showing such features as neurodegeneration and an increased level of Akt and oxidative stress [94]. Genetic studies on *C. elegans* have uncovered several genes associated with manganese neurotoxicity. Furthermore, a significant number of studies have been conducted to decipher the role of astrocytes in manganese-induced neurotoxicity [95,96]. The use of a simple organism such as *C. elegans* that has homologous human genes has recently attracted attention for defining and discovering the exact pathway involved in manganese uptake/transport and explaining the underlying pathophysiology [97,98].

### 5.1. Disorders Associated with Manganese Homeostasis

#### 5.1.1. Hypermanganesemia with Dystonia Type 1

Hypermanganesemia with dystonia type 1 (OMIM 613280) is a disorder associated with improper manganese homeostasis caused by homozygous mutations in the *SLC30A10* gene (OMIM 611146) located on chromosome 1q41 [99,100]. *SLC30A10* (Solute carrier family 30 member 10) encodes for a manganese exporter that is expressed in the liver, small intestine, and brain [101,102]. To date, only 22 mutations in the *SLC30A10* gene causing hypermanganesemia with dystonia type 1 have been reported, which mostly include small deletions (HGMD, 2018; http://www.hgmd.cf.ac.uk).

#### 5.1.2. Hypermanganesemia with Dystonia Type 2

Pathogenic mutations in the *SLC39A14* gene located on chromosome 8p21.3, which encodes for ZIP14, have been associated with two different disorders [103]. Heterozygous mutations in the dominant fashion are responsible for hyperostosis cranalis interna (OMIM 144755), while biallelic mutations have been reported to cause hypermanganesemia with dystonia 2 (OMIM 617013).

The associated phenotypes includes microcephaly, bulbar dysfunction, scoliosis, axial hypotonia, intellectual disability, variable learning disability, poor or absent speech, dystonia, spasticity, abnormal gait, scissoring, and hyper-reflexia. A decrease in the uptake of manganese has been observed in several patients; thus, the liver is safe, while an elevation of manganese concentration in the blood has been observed that leads to manganese deposition in the brain [103].

The *slc39a14* zebrafish model showed a buildup of manganese in the brain, while no other metals were accumulated that were associated with reduced locomotion activity. The main issue was the proper uptake of manganese into the liver, which usually allows the blood manganese level to be normalized via biliary excretion [103].

#### 5.1.3. Congenital Disorder of Glycosylation Type IIn

Homozygous mutations in the *SLC39A8* gene (OMIM 608732) located on chromosome 4q24 have been associated with congenital disorder of glycosylation type IIn (OMIM 616721). Clinical features associated with the disorder include short stature, eye deformity (strabismus, astigmatism, nystagmus), osteopenia, hypotonia, an inability to walk, and laboratory results suggested decreased plasma zinc and manganese levels and increased urinary zinc and manganese levels [104,105].

## 6. Selenium Homeostasis

The total selenium intake in the human body varies across different countries. In the United States, the adult intake level is 13.0–20.3 mg [106]. People living in other countries have a low intake of selenium. It is mostly found in an organic form in plant sources (selenomethionine) and animal sources (selenocysteine). In dietary supplements, it is found in inorganic forms (selenite and selenite) [107].

Selenium is mostly required for the proper activity and biosynthesis of selenoproteins. By definition, a selenoprotein is a protein with a selenocysteine (Sec, U, Se-Cys) amino acid residue. Almost 25 genes in the human genome that encode for different types of selenoproteins have been identified [106,108]. The primary functions of different mammalian selenoproteins are presented in Table 7. The intestine absorbs approximately 80% of ingested selenium [108,109,110,111]. Different disorders have been reported to be associated with mutations in the genes encoding for these selenoproteins. They are usually very complex syndromes [107].

### 6.1. Disorders Associated with Manganese Homeostasis

#### 6.1.1. Keshan Disease

Keshan disease is a severe disorder that exhibits an endemic cardiomyopathy and was reported for the first time in selenium-deficient areas of China [112]. Multifocal myocarditis was observed primarily in children aged 2–10 years and, to a lesser extent, in women of childbearing age [113,114]. Its main manifestations are cardiac enlargement, insufficiencies in cardiac function, electrocardiographic abnormalities, arrhythmias, and radiographic abnormalities [112].

#### 6.1.2. Rigid Spine Muscular Dystrophy 1 (RSMD1) and Congenital Myopathy with Fiber-Type Disproportion

Rigid spine muscular dystrophy-1 (OMIM 602771), desmin-related myopathy with Mallory bodies, congenital myopathy with fiber-type disproportion (OMIM 255310), and severe classic multiminicore myopathy have the same severe disease spectrum and are caused by a pathogenic biallelic or compound heterozygous mutation in the *SEPN1* gene (OMIM 606210) located on chromosome 1p36. The *SEPN1* gene is abbreviated as *SELENON* (Selenoprotein N) and encodes for a particular glycoprotein that is mostly localized in the endoplasmic reticulum, which plays a key role in the regulation of calcium homeostasis and protection against oxidative stress [115,116,117].

RSMD1 is inherited as an autosomal recessive entity. Clinical features associated with RSMD1 include short stature, low body weight, poor ability to gain weight, poor head control, failure to thrive, restrictive respiratory syndrome, reduced vital capacity, spinal rigidity, hypotonia, and other associated severe conditions [115,116,117,118].

Congenital myopathy with fiber-type disproportion (CFTD) is inherited in both an autosomal dominant and a recessive fashion. Clinical features associated with CFTD include failure to thrive, a long face, facial muscle weakness, ophthalmoplegia (in 20% of patients), ptosis, respiratory distress, decreased forced vital capacity, neonatal hypotonia, proximal muscle weakness, and some other associated conditions [115,118].

#### 6.1.3. Abnormal Thyroid Hormone Metabolism or SECISBP2 Syndrome

Homozygous or compound heterozygous mutations in the *SECISBP2* gene (OMIM 607693) have been associated with abnormal thyroid hormone metabolism (OMIM 609698). This syndrome is associated with such features as short stature, soft tissue weakness, proximal muscle weakness, and infiltration of fat into muscles. Laboratory tests suggest an increase in serum thyrotropin, total thyroxine (T(4)), free thyroxine (T(4)), and reverse triiodothyronine (rT(3)), while triiodothyronine (T(3)) and serum selenium levels are decreased in the body [119,120]. To date, only 15 mutations causing abnormal thyroid metabolism, growth retardation, and selenoprotein-related pathogenesis have been associated with the *SECISBP2* gene (HGMD, 2018; http://www.hgmd.cf.ac.uk).

#### 6.1.4. Pontocerebellar Hypoplasia Type 2D

Pontocerebellar hypoplasia (PCH2D; OMIM 613811) is a rare heterogeneous neurodegenerative disorder inherited as an autosomal recessive entity. Homozygous mutations in the *SEPSECS* gene (OMIM 613009) have been reported to cause this severe condition, which is characterized by such features as microcephaly, contractures, an intellectual disability, clonus, seizures, sleep disturbances, cerebellar atrophy, and thin corpus callosum [121,122,123]. To date, only 16 mutations causing pontocerebellar hypoplasia have been associated with the *SEPSECS* gene (HGMD, 2018; http://www.hgmd.cf.ac.uk).

#### 6.1.5. Glutathione Peroxidase Deficiency

Glutathione peroxidase deficiency (GPXD; OMIM 614164), in association with hemolytic anemia, has been reported several times in the literature. It is an autosomal recessive disorder with such hallmark clinical features as neonatal hyperbilirubinemia, interaction with selenium deficiency, hemolytic issues, compensated hemolytic anemia, and laboratory tests that reveal glutathione peroxidase deficiency and heinz bodies [124,125,126]. Mutations in the *GPX1* gene (OMIM 138320), which is located on chromosome 3p21.31, have been associated with GPXD [127].

#### 6.1.6. Sedaghatian-Type Spondylometaphyseal Dysplasia (SMDS)

SMDS (OMIM 250220) is a lethal disorder clinically characterized by platyspondyly, severe metaphyseal chondrodysplasia, delayed epiphyseal ossification, limb shortening, pulmonary hemorrhage, and irregular iliac crests. Affected individuals show severe hypotonia and cardiovascular and respiratory problems [128,129]. Due to respiratory failure, most patients die within a few days after birth [130]. It is an autosomal recessive disorder and compound heterozygous mutations in the *GPX4* gene (OMIM 138322), which is located on chromosome 19p13.3, have been associated with SMDS [131]. GPX4 (glutathione peroxidase 4) belongs to the glutathione peroxidase family. Its isozyme is also a selenoprotein that contains the rare amino acid selenocysteine (Sec) at its active site, which is involved in catalyzing the reduction of hydrogen peroxide, lipid hydroperoxides, and organic hydroperoxides and, thus, protecting cells against oxidative damage [132].

## 7. Treatment for Metal-Associated Disorders

Disorders associated with transition metals can be effectively treated and the metal deficiency may be fulfilled by oral supplements. Treatment for metal toxicity can also be accomplished by using another metal that competes for the transport or uptake of the desired one; for example, for intestinal uptake or transport, iron competes with magnesium (DMT1). Thus, transferrin/a transferrin receptor can be utilized in those disorders where manganese accumulates [133]. Chelation therapy is also used as an alternative metal toxicity treatment. For instance, the chelation of copper is performed with such agents as trientine and penicillamine. As a treatment for disorders associated with manganese toxicity, intravenous infusion of disodium calcium edetate can be performed effectively [99,103,133].

Two main factors should be kept in mind while using a chelator. The first is the stability constant, as disodium calcium edetate will only chelate metals that have the capacity to bind a higher-stability constant, such as calcium (Mn, Fe, Co, Zn, Cd, Pb, Ni). Secondly, disodium calcium edetate will bind much more efficiently to unbound metal ions as compared to bound ones [103].

Chelators and ionophores target transition metal homeostasis at the molecular level by binding and releasing metals with the aim of eliminating excess metals, depositing exogenous metals, or redistributing endogenous metals. Ionophores and chelators may work opposite one another, as ionophores are responsible for the delivery of metals and chelators remove metals. Ultimately, they both act as metal-binding molecules [6]. Chelators and ionophores have application as metal-binding molecules in different diseases, including cancer [134,135] and neurodegenerative diseases [6,136]. Researchers are developing chelators and ionophores for drugs that target metal homeostasis, and these chelators and ionophores are making their way into clinical trials for the treatment of cancer and neurodegenerative diseases [137,138].

## 8. Conclusions

Metabolic disorders related to metals are regarded as quintessential single-gene disorders. However, in recent years, advances in next-generation sequencing and other advanced technologies have revealed that the classical "one gene–one enzyme" paradigm is not always the culprit. It has been reported that defects in multiple genes may lead to the same phenotypic presentation. Furthermore, mutations in different domains of the same protein might produce a difference in the phenotypes. Thus, it very difficult to pin-point a specific mutation and proceed with therapeutic strategies as different mutations respond differently during therapy. This process is not homogeneous across diseases and specific mutations.

In addition, next-generation sequencing has also shown that affected individuals with hybrid phenotypes might have mutations in more than two different genes that are responsible for causing more than two phenotypes in the same individual, which leads to a complex phenotype. Such complex genetic disorders are very difficult to diagnose and become increasingly difficult to manage with a therapeutic plan. Newly discovered disease-causing genes and disorders associated with metal metabolism have provided us with insight into the basics of the micronutrients that are required for the proper maintenance of the human body. Advances in the fields of molecular diagnostics and precision medicine have allowed us to understand more about the metabolism, regulation, and functions of these micronutrients, which will ultimately lead to new therapeutic strategies. However, we still have a lot to learn.

## Figures and Tables

**Table 1:** Disorders that are due to an accumulation of manganese, copper, or iron and cause neurotoxicity.

Disorders	Transition Metal	Inheritance	Gene	OMIM	Gene Function	Symptoms
Hypermanganesemia with dystonia 1	Manganese	Autosomal recessive	*SLC30A10*	613280	Manganese transporter	Dystonia, cock-walk gait Parkinsonism
Hypermanganesemia with dystonia 2	Manganese	Autosomal recessive	*SLC39A14*	617013	Manganese transporter	Progressive dystonia and bulbar dysfunction
Wilson’s disease	Copper	Autosomal recessive	*ATP7B*	277900	Copper transporter	Dysarthria, dysphagia, tremor, dystonic rigidity
Acaeruloplasminaemia, Cerebellar ataxia, Hypoceruloplasminemia	Iron	Autosomal recessive	*CP*	604290	Ferroxidase	Chorea, ataxia, dystonia, Parkinsonism, Diabetes mellitus
Neuroferritinopathy, Hyperferritinemia-cataract syndrome, L-ferritin deficiency	Iron	Autosomal dominant	*FTL*	600886 615604 606159	Iron storage	Chorea, dystonia
Spastic paraplegia type 35	Iron	Autosomal recessive	*FA2H*	612319	Fatty acid 2-hydroxylase (Synthesis of sphingolipids)	Gait difficulties with spastic paraparesis and dysmetria
Neurodegeneration with brain iron accumulation 1, HARP syndrome	Iron	Autosomal recessive	*PANK2*	607236 234200	Pantothenate kinase (CoA synthesis)	Dystonia, rigidity, choreoathetosis
Neurodegeneration with brain iron accumulation 6, Pontocerebellar hypoplasia type 12	Iron	Autosomal recessive	*COASY*	615643 618266	CoA synthesis	Dystonia, rigidity, choreoathetosis
Infantile neuroaxonal dystrophy 1, Neurodegeneration with brain iron accumulation 2B, Parkinson’s disease type 14	Iron	Autosomal recessive	*PLA2G6*	256600 610217 612953	Phospholipase	Motor regression, hypotonia
Spastic paraplegia 43, Neurodegeneration with brain iron accumulation 4	Iron	Autosomal recessive	*C19orf12*	615043 614298	Mitochondrial magnesium homeostasis	Spastic paraplegia, Parkinsonism
Woodhouse–Sakati syndrome	Iron	Autosomal recessive	*DCAF17*	241080	Ubiquitinylation	Developmental delay Dystonia, dysarthria, choreoathetosis
Neurodegeneration with brain iron accumulation type 5	Iron	X-linked dominant	*WDR45*	300894	Autophagy	Global developmental Delay, Delayed psychomotor development, Mental retardation, Poor speech, Lack of speech
Kufor–Rakeb syndrome, Spastic paraplegia type 78	Iron	Autosomal recessive	*ATP13A2*	606693 617225	Lysosomal divalent cation (transition metal) transporter	Atypical Parkinsonism Supranuclear gaze palsy Spasticity

**Table 2:** Disorders associated with iron metabolism leading to hepcidin deficiency.

S. No.	Gene	Inheritance	Protein	Role in Fe Metabolism	Anaemia	Treatment
1	*HAMP* *(606464)*	Autosomal recessive	Hepcidin	Inhibits iron release by ferroportin	-----	Not Available
2	*HFE1* *(235200)*	Autosomal recessive	HFE	Regulates synthesis of hepcidin	-----	Phlebotomy for ferritin >1000 ng/mL
3	*HJV* *(608374)*	Autosomal recessive	Haemojuvelin	Regulates synthesis of hepcidin	-----	Phlebotomy effective if started early
4	*TRF2* *(602027)*	Autosomal recessive	Transferrin receptor 2	Regulates synthesis of hepcidin	-----	Phlebotomy
5	*SLC40A1* *(604653)*	Autosomal dominant	Ferroportin (loss of function)	Export from cells into circulation	Mild microcytic	Phlebotomy
			Ferroportin (gain in hepcidin resistance)			

**Table 3:** A list of genes involved in neurodegeneration with iron accumulation in the brain (NBIA).

S. No.	Gene (OMIM)	Inheritance	Protein	Disorder (OMIM)	Role in Fe Metabolism	Other	Treatment
1	*SLC11A2* *(600523)*	Autosomal recessive	DMT1	Anemia, hypochromic microcytics with iron overload 1 (206,100)	Duodenal uptake Intracellular release	Increased Cu in brain associated with impulsivity in a rat model	Erythropoietin (EPO)
2	*TMPRSS6* *(609862)*	Autosomal recessive	Matriptase 2	Iron-refractory, iron deficiency anemia (206,200)	Mutations lead to high hepcidin levels	-----	-----
3	*STEAP3* *(609671)*	Autosomal dominant	STEAP3	Anemia, hypochromic microcytics with iron overload 2 (615,234)	For cytoplasmic uptake endosomal ferrireductase is required	Hepatosplenomegaly Hypopituitarism Hypogonadism	Transfusion Fe chelation
4	*TFRC* *(190010)*	Autosomal recessive	Transferrin receptor 1	Immunodeficiency 46 (616,740)	Cellular uptake	Combined immunodeficiency Leukopenia Thrombocytopenia	-----
5	*TF* *(190000)*	Autosomal recessive	Transferrin	Atransferrinemia (209,300)	Fe transport in blood, uptake	Growth retardation (TF < 20 mg/dL)	Plasma infusions

**Table 4:** The copper balance in the body [4,44,45].

S. No.	Copper Amount	Wilson Disease	Normal	Menkes Disease
1	Copper dietary intake (mg/day)	5	5	5
2	Copper intestinal absorption (mg/day)	2	2	0.1–0.2
3	Copper biliary excretion (mg/day)	0.2–0.4	2	------
4	Copper urinary excretion (ug/day)	100–1000	15–60	increased
5	Copper balance	Positive	0	Negative
6	Serum copper (mg/L)	0.19–0.63	0.75–1.45	<0.70
7	Serum ceruloplasmin (mg/L)	0–200	180–360	<50
8	Liver copper (ug/g dry weight)	200–3000	70–140	10–20
9	Duodenal copper (ug/g dry weight)	------	7–29	50–80

**Table 5:** List of enzymes containing copper.

S. No.	Enzyme	Function	Deficiency
1	Sulfhydryl oxidase	Cross-linking of keratin	Pili torti
2	Diamine oxidases	Degrade histamine	Histamine response increased
3	Superoxide dismutase	Free radical detoxification	Protection against oxygen free radicals decreased
4	Peptidylglycine-aminating monoxygenase	Removes carboxy-terminal glycine to activate neuroendocrine peptides	Reduced activity of gastrin, cholecystokinin
5	Lysyl oxidase	Cross-linking of collagen and elastin	Arterial abnormalities; bladder diverticulae; lax skin and joints
6	Dopamine-β-hydroxylase	Dopamine production in neurons Catecholamine production	Reduced number of neurotransmitters, Neurologic effects; temperature instability; pupillary constriction
7	Tyrosinase	Melanin production in skin	Reduced pigmentation
8	Cytochrome c oxidase	Electron transport chain	Decreased energy in muscle and neurons; impaired myelination;
9	Ascorbate oxidase	Dehydroascorbate production	Skeletal demineralization
10	Ceruloplasmin	Fe (II)/Fe (III) oxidation and may be involved in lipid peroxidation	Aceruloplasminemia, Cerebellar ataxia, Hemosiderosis
11	Hephaestin	Intestinal iron efflux	Hemochromatosis Type 1 Deficiency, Anemia
12	Zyklopen	Placental iron efflux	Abnormal hair, joint laxity, and developmental delay

**Table 6:** Enzymes that require manganese for proper function [4].

S. No.	Enzyme	Function
1	Prolidase	Collagen recycling (Enzyme capable of degrading dipeptides)
2	Arginase	Krebs-Henseleit urea cycle (Final enzyme of the urea cycle)
3	Superoxide dismutase	Antioxidant (An enzyme that alternately catalyzes the dismutation of the superoxide radical into either ordinary molecular oxygen or hydrogen peroxide)
4	Glycosyl tranferases	Glycosaminoglycan synthesis (Enzymes that establish natural glycosidic linkages)
5	Glutamine synthetase	Glutamine synthesis (Plays an essential role in the metabolism of nitrogen by catalyzing the condensation of glutamate and ammonia to form glutamine)
6	Isocitrate dehydrogenase	Krebs cycle (Catalyzes the oxidative decarboxylation of isocitrate, producing alpha-ketoglutarate and CO₂)
7	Pyruvate carboxylase	Gluconeogenesis (It catalyzes the physiologically irreversible carboxylation of pyruvate to form oxaloacetate)
8	Phosphoenolpyruvate carboxykinase	Gluconeogenesis (It converts oxaloacetate into phosphoenolpyruvate and carbon dioxide)

**Table 7:** Mammalian selenoproteins and their function [107,110,111].

Mammalian Selenoproteins	Functions
15 kDa selenoprotein (Sep15)	Regulated by ER stress, interacts with the UDP-glucose glycoprotein glucosyltransferase and is potentially involved in glycoprotein folding
Thyroid hormone deiodinase 1	Removes iodine from the outer ring of T4 to produce plasma T3 and catalyzes deiodination and thus inactivation of T3
Thyroid hormone deiodinase 2	Converts T4 to T3 locally in tissues
Thyroid hormone deiodinase 3	Catalyzes deiodination of T4 to T3 in peripheral tissues
Glutathione peroxidase (GPx) 1–4, 6	GSH-dependent detoxification of H2O2 (enriched in liver, kidney, erythrocytes)
Selenoprotein H	Nuclear redox control, protects cells from H2O2, increases mitochondrial biogenesis and CytC production
Selenoprotein I	Unknown function
Selenoprotein R (SEPX, MSRB1)	Methionine sulfoxide reductase
Selenoproteins K	Modulates Ca2+ influx that affects immune cells
Selenoproteins M	Protects neurons from oxidative stress
Selenoproteins N,	Expressed in skeletal muscle, heart, lung, and placenta
Selenoproteins, O	Unknown function
Selenoproteins, T	Cellular calcium handling, redox regulation, plays a role in cell adhesion
Selenoprotein, P (SelP)	Se transport to peripheral tissues and an antioxidant function
Selenoprotein, R	Reduces methionine-R-sulfoxide residues in proteins to methionine
Selenoproteins, S	Upregulated upon treatment with pro-inflammatory cytokines and glucose deprivation Protein folding and degradation
Selenophosphate synthetase 2 (SPS2)	Selenocysteine biosynthesis
Selenoprotein P (SePP)	Se transport
Deiodinase (DIO) 1–3	Thyroid hormone metabolism
Thioredoxin reductase 1	Reduces the oxidized form of cytosolic thioredoxin
Thioredoxi	Catalyzes a variety of reactions, specific for thioredoxin and glutaredoxin systems, and expressed in spermatids
Thioredoxin reductase 3	Reduces the oxidized form of mitochondrial thioredoxin and glutaredoxin 2
Selenoprotein, V	Unknown function and expressed in spermatids
Selenoprotein W	Unknown function and expressed in skeletal muscle and other tissues
